# Multilevel analysis of variance used to partition components of optical imprecision in an analyser system with disposable cuvettes

**DOI:** 10.1155/S1463924681000060

**Published:** 1981

**Authors:** Douglas M. Fast, Eric J. Sampson, Carl A. Burtis

**Affiliations:** 1 Clinical Chemistry Division Bureau of Laboratories Center for Disease Control, Public Health Service U.S. Department of Health and Human Services Atlanta Georgia 30333 USA; 2 Chemical Technology Division 4500N Oak Ridge National Laboratories Oak Ridge TN 37830 USA


ACKNOWLEDGEMENTS
The authors thank the Department of Health and Social Security for providing the system to be tested, Dr Burnett of St Albans City Hospital and Dr Cheng of Watford General Hospital for their help with the comparative studies and the staff of Instrumentation Laboratory Ltd for their co-operation. The advice of Dr S S Brown of the Clinical Research Centre, Harrow and Dr I W Percy-Robb of Edinburgh Royal Infirmary is also gratefully acknowledged.

REFERENCES
[1] Gibson P F, Calzi C, Musetti A, and Caliri R. X International Congress of Clinical Chemistry, Mexico City, 1978.
[2] Broughton P M G, Gowenlock A H, McCormack J J, and Neill D W (1974) *Annals of Clinical Biochemistry,* **11**, 207-218.

### Multilevel analysis of variance used to partition components of optical imprecision in an analyser system with disposable cuvettes*

**Douglas M. Fast, Eric J. Sampson, Carl A. Burtis****
*Clinical Chemistry Division, Bureau of Laboratories, Center for Disease Control, Public Health Service, U.S. Department of Health and Human Services, Atlanta, Georgia 30333, USA.*

### Introduction
With the increasing use of disposable cuvettes in modern spectrophotometric instrumentation it is vital for the analyst to be aware of the various types of errors that can be introduced into the analytical process. Other investigators have described these errors and their propagation in spectrometric systems [1-5] or have examined random errors in various specific components of their systems [6-10]. On the basis of these studies, various professional organisations have proposed guidelines for spectrometric instruments [11-14]. However, for an analytical system using a disposable rotor containing a large number of cuvettes which is used only once and discarded, a statistical technique must be implemented to quantitate random optical error, to check actual instrument performance against manufacturer's specifications, to assess the quality of incoming supplies for the centrifugal analyser, and to provide criteria for explicit operational practices in the use of the analyser.

Analysis of variance (ANOVA) has previously been used in assigning magnitudes of error to sources within a multiunit instrument or method [15], but has been usually limited to an examination of only two or three variables. This approach has been extended to a three-level nested ANOVA which separates the random optical noise component from errors in absorbance associated with possible changes in the physical parameters of the disposable cuvettes. These errors include variations in the absorbance within-cuvettes, between-cuvettes, between rotors, and between manufacturer's lots of rotors.

### Materials and methods
### *Instrument*
The centrifugal analyser system evaluated was the Multistat III micro centrifugal analyser [3] (Instrumentation Laboratory, Lexington, MA 02173 USA). The analyser system consists of two modules, an automated loader and the spectrophotometric analyser. The analyser uses disposable plastic cuvettes (20 per rotor) for incubation, mixing, and measurement of absorbance (Figure 1). The 0.5 cm optical cell is formed by moulding clear windows in the cuvette top and bottom surfaces. Of the 20 cuvettes in the rotor, the first is used as the reference cuvette, and the remaining 19 are used for any combination of standards and samples necessary. Narrow bandpass interference filters are used in the photometer to isolate the spectral range of interest. Transmitted radiation is measured by using a photomultiplier tube and an

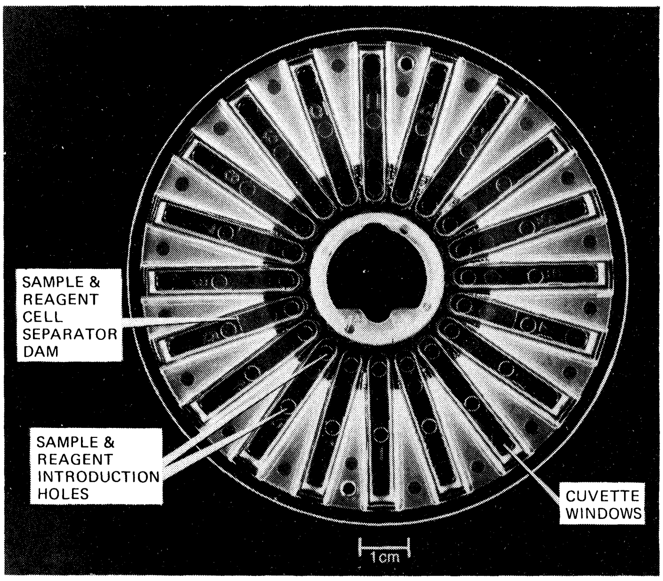

*Figure 1. Disposable rotor used with Multistat III analyser system (top view). Cuvette windows are an integral part of the top and bottom walls of each cuvette. Upon acceleration, sample flows over the separator dam and mixes with reagent to initiate chemical reaction.*

*Presented in part at the Pittsburgh Conference on Analytical Chemistry and Applied Spectroscopy, Cleveland, Ohio, 1979.*
***Present address: Chemical Technology Division, 4500N, Oak Ridge National Laboratories, Oak Ridge, TN 37830.*

autoranging gain circuit so that the signal from the reference cuvette is always set to yield the maximum output from the analog-to-digital converter no matter how much the intensity of the reference beam varies as different filters are used. This study was concerned only with the photometric module error; consequently, no evaluation of the loader module was made. Photometric performance was evaluated at the wavelength most used in this laboratory, 340 nm.

### *Reagents and solutions*

The solution whose absorbance was measured at 340 nm was prepared by dissolving 19.4 mg of B-Nicotinamide-adenine dinucleotide, reduced, disodium salt (NADH) (Grade II, Boehringer Mannheim Biochemicals, Indianapolis, IN 46250 USA) in 200 ml of 0.1 mol/l, ph 7.8, tris(hydroxy-methyl) aminomethane hydrochloride (Tris) buffer (Sigma Chemical Company, St. Louis, MO 63178 USA). The NADH solution was freshly prepared each day, protected from light, and stored at 4°C when not in use.

### *Experimental design*

Three rotors from each of four rotor lots were randomly selected (Figure 2). Deionised-distilled water was used as the reference in cuvette number one of each rotor. The remaining 19 cuvettes were hand loaded (SMI Micro/pettor, Scientific Manufacturing Industries, Berkeley, CA 94710 USA) with 250 $\mu$L of the prepared NADH solution. The rotors were then placed in the analyser and allowed to equilibrate for 5 minutes at 30°C. The absorbance at 340 nm in each cuvette (approximately 0.4) was reported 12 times at 5-second intervals. Each of the 12 absorbances reported was itself the mean value of 32 individual samplings of the transmitted energy level.

### Mathematical model

Any single absorbance measurement ($y_{ijkl}$) can be described as

$$y_{ijkl} = \mu + L_i + R_{i(j)} + CU_{i(jk)} + \epsilon_{ijkl} \quad (1)$$

where $\mu$ is the true value of the absorbance, $L_i$ is the lot effect, $R_{i(j)}$ is the rotor-within-lot effect, $CU_{i(jk)}$ is the cuvette-within-rotor-within-lot effect, and $\epsilon_{ijkl}$ is the random error associated with the ijkl-th measurement. The number of lots (4), rotors in a lot (3), cuvettes in a rotor (19), and measurements in a cuvette (12) are assigned as $n_1$, $n_2$, $n_3$, and $n_4$, respectively. The total number of measurements made, N, is defined as:

$$N = (n_1)(n_2)(n_3)(n_4) \quad (2)$$

A corrected mean (CM) is defined [15] as:

$$CM = \frac{\left[\sum_{i=1}^{n_1} \sum_{j=1}^{n_2} \sum_{k=1}^{n_3} \sum_{l=1}^{n_4} y_{ijkl}\right]^2}{N} \quad (3)$$

Measurements are further classified into cells as follows:

$$RDG_{ijk} = \sum_{l=1}^{n_4} y_{ijkl} \quad (4)$$

$$CU_{ij} = \sum_{k=1}^{n_3} RDG_{ijk} \quad (5)$$

and

$$ROT_i = \sum_{j=1}^{n_2} CU_{ij} \quad (6)$$

The total sum of squares, SST, is defined as:

$$SST = SSL + SS(ROT\ in\ L) + SS(CU\ in\ ROT) + SS(RDG\ in\ CU) \quad (7)$$

where SSL is the sum of squares due to the lots, SS(ROT in L) is the sum of squares due to the rotors in the lots, SS(CU in ROT) is the sum of squares due to the cuvettes in the rotors, and SS(RDG in CU) is the sum of squares due to readings in the cuvettes.

The ANOVA table can now be written as shown in Table 1 where CM, $ROT_i$, $CU_{ij}$, and $RDG_{ijk}$ are as defined in Equations 3 - 6. Assuming a random effects model, the expected mean squares (EMS) are then as shown in Table 2, where $\sigma_\epsilon^2$ is the variance of the mean of the 32 samplings comprising one reading, $\sigma_{CU}^2$ is the variance due to between-cuvette error, $\sigma_{ROT}^2$ is the variance between rotors in a lot, and $\sigma_L^2$ is the variance between lots of rotors [15]. Estimated values for $\sigma_\epsilon^2$ (the variance of the mean of the samplings) were multiplied by the number of individual measurements per reported mean absorbance (i.e., 32) to estimate the variance, $\sigma^2$, of a single absorbance determination made on the instrument [16]. This variance, $\sigma^2$, should be considered the true "noise" of the photometer system for a single (not a reported) absorbance determination. This ANOVA scheme was implemented in a FORTRAN program which may be obtained from the authors.

### Results and Discussion

Table 3 shows the results of the three-level nested ANOVA performed on 2736 absorbance measurements. The grand mean was an absorbance (A) of 0.4194. The null hypotheses that variances $\sigma_{CU}^2$, $\sigma_{ROT}^2$, and $\sigma_L^2$ equal zero were rejected ($\alpha$ = 0.001) by F-testing, giving statistical evidence of variation between lots, between rotors within lots, and between cuvettes within rotors. The mean square value for "Readings-within-Cuvettes" was calculated as 5.18 x $10^{-6}$ (Table 3). The previous description of the mathematical model showed that this computed value is really the variance of a mean of 32 measurements ($s_{\bar{x}}^2$). The "noise" of the photometer unit (i.e., the expected variance for a single photometric sampling) is 1.66 x $10^{-4}$ (SD = 1.29 x $10^{-2}$A, CV = 3.1%). Though the SD of the single sampling (12.9 mA) seems relatively large, the absorbance value reported to the user is in fact the mean of 32 of these readings. Because of this averaging, the mean value reported will provide a 95% confidence interval of ± 4.5 mA. Table 3 now shows that the total variance is then partitioned as follows: between-lot (46.2%); readings-within-cuvettes (40.3%); between-cuvette (9.8%); and between-rotor (3.7%). If one assumes that

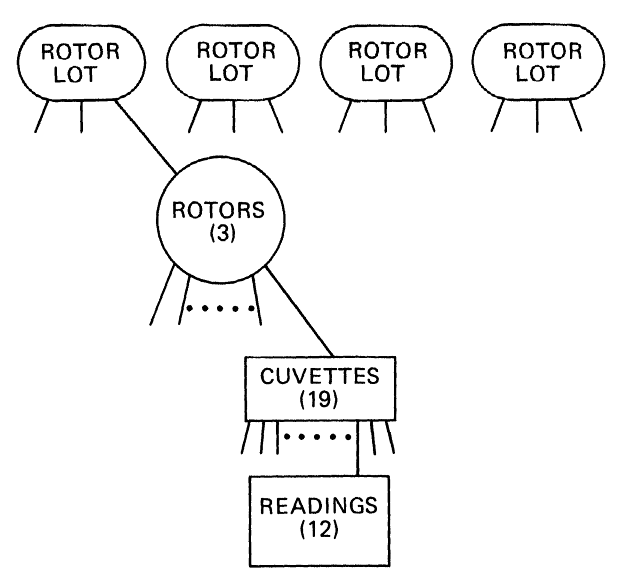

*Figure 2. Design of Experiment. Three rotors were sampled randomly from four lots. Twelve measurements were made in each of the 19 cuvettes per rotor.*

**Table 1. Three-level nested, balanced design ANOVA.**

| Source | df | Sum of squares | Mean square |
|---|---|---|---|
| Lots | $n_1 - 1$ | $\dfrac{\sum_{i=1}^{n_1} ROT_i^2}{n_2 n_3 n_4} - CM \equiv SSL$ | $SSL/(n_1 - 1)$ |
| Rotors within lots | $n_1(n_2 - 1)$ | $\dfrac{\sum_{i=1}^{n_1}\sum_{j=1}^{n_2} CU_{ij}^2}{n_3\ n_4} - \dfrac{\sum_{i=1}^{n_1} ROT_i^2}{n_2 n_3 n_4} \equiv SS(ROT\ in\ L)$ | $\dfrac{SS\ (ROT\ in\ L)}{n_1\ (n_2 - 1)}$ |
| Cuvettes within rotors | $n_1 n_2\ (n_3 - 1)$ | $\dfrac{\sum_{i=1}^{n_1}\sum_{j=1}^{n_2}\sum_{k=1}^{n_3} RDG_{ijk}^2}{n_4} - \dfrac{\sum_{i=1}^{n_1}\sum_{j=1}^{n_2} CU_{ij}^2}{n_3 n_4} \equiv SS\ (CU\ in\ ROT)$ | $\dfrac{SS(CU\ in\ ROT)}{n_1 n_2\ (n_3 - 1)}$ |
| Readings within cuvettes | $n_1 n_2 n_3 (n_4 - 1)$ | $\sum_{i=1}^{n_1}\sum_{j=1}^{n_2}\sum_{k=1}^{n_3}\sum_{\ell=1}^{n_4} y_{ijk\ell}^2 - \dfrac{\sum_{i=1}^{n_1}\sum_{j=1}^{n_2}\sum_{k=1}^{n_3} RDG_{ijk}^2}{n_4} \equiv SS\ (RDG\ in\ CU)$ | $\dfrac{SS\ (RDG\ in\ CU)}{n_1 n_2 n_3\ (n_4 - 1)}$ |
| TOTAL | $n_1 n_2 n_3 n_4 - 1$ | $\sum_{i=1}^{n_1}\sum_{j=1}^{n_2}\sum_{k=1}^{n_3}\sum_{\ell=1}^{n_4} y_{ijk\ell}^2 - CM$ | |

these four variances are additive [17], the overall standard deviation (SD) of a single photometric sampling is an absorbance of 0.0203 and the coefficient of variation (CV) is 4.8%.

Further treatment of the data by a one-way ANOVA on each rotor in turn disclosed that the three rotors from one lot had within-cuvette $s_{\bar{x}}$ values of 0.705, 2.334, and 7.345 mA. On visual examination, rotors taken from the lot with the largest $s_{\bar{x}}$'s were found to contain particulate material inside the cuvettes. On the basis of this finding, operators of the system have been instructed to ascertain that cuvettes and the cell windows are free from particulate contamination before using a rotor. However, subsequent lots of rotors have not appeared to have this contamination problem.

Eight of the nine rotors from the remaining three lots had within-cuvette $s_{\bar{x}}$ values of $< 0.53$ mA and the ninth had a within-cuvette $s_{\bar{x}}$ of 1.03 mA. This data was reanalysed by using the three-level nested ANOVA (Table 4). The overall coefficient of variation of the optical unit was 3.4% of the 0.4144 absorbance grand mean (n = 2052). The between-lot variance accounted for 68.6% of the total variance; the between-rotor variance, for 10.2% of the total variance; and the between-cuvette variance, for 17.2% of the total; but the variance component attributed to a single photometric sampling decreased from 40.3% to 4.0% of the total. The SD of a single photometric reading was now 2.82 mA, a decrease from the 12.9 mA computed for all 12 rotors (Table 3). The between-cuvette SD was 5.9 mA. The linear combination for the four variance components [17] indicated that the overall SD of a single photometric sampling using these three lots of rotors was an absorbance of 0.0142 (CV = 3.4%). The null hypothesis of no variation between lots was accepted ($\alpha = 0.001$), but similar hypotheses of no variation between rotors in lots and of no variation between cuvettes in rotors were rejected ($\alpha = 0.001$). The effects of changing rotors and lots may be a realistic estimate of the between-day precision available for equilibrium measurements with this instrument.

Tiffany et al. [5] reported an uncertainty of 0.14 mA at an absorbance of 0.44 for a prototype Multistat III and they calculated that the worst-case photometric imprecision was about 2.5% (CV) for an actual enzyme measurement. It is clear from the description of their experiment that this statistical uncertainty is the standard error of the mean ($s_{\bar{x}}$) for the absorbance values reported. To compare the prototype instrument with our production model, their standard error at 0.4144 absorbance can be squared and compared with the within-cuvette mean square value in our Table 4. In doing this computation, it is found that a mean square of 25 x $10^{-8}$ is an order of magnitude greater than the value of approximately 2 x $10^{-8}$. Likewise from the data given in Figure 2 of Tiffany, et al. [5] for runs reporting the mean absorbance of 36 photometric samplings, one can calculate (at an absorbance of 0.4144) a single sampling variance of $(7.2 \times 10^{-5}\ e^{1.69A})^2 \times 36 = 7.6 \times 10^{-7}$ (SD = 0.9 mA). The variance computed for the instrument for a single sampling (Table 4) is 8.0 x $10^{-6}$ (SD = 2.8 mA), again about an order of magnitude greater than the prototype model.

Maclin [8] has shown that for a GEMSAEC centrifugal analyser the standard error of the mean absorbance versus the measured absorbance is given by the equation $A = 0.000072e^{1.597A}$. At an absorbance of 0.4144, his

**Table 2. Three-level nested, balanced design expected mean squares.**

| Source | Expected mean squares |
|---|---|
| Lots | $\sigma_\epsilon^2 + n_4\ \sigma_{CU}^2 + n_3 n_4\ \sigma_{ROT}^2 + n_2 n_3 n_4\ \sigma_L^2$ |
| Rotors within lots | $\sigma_\epsilon^2 + n_4\ \sigma_{CU}^2 + n_3 n_4\ \sigma_{ROT}^2$ |
| Cuvettes within rotors | $\sigma_\epsilon^2 + n_4\ \sigma_{CU}^2$ |
| Readings within cuvettes | $\sigma_\epsilon^2$ |

**Table 3. Three-level nested, balanced design ANOVA – 4 Lots.**

| Source | df | Sum of squares | Mean square | Mean square ratios | Variance | % of total variance | Standard deviation (mA) | CV(%) |
|---|---|---|---|---|---|---|---|---|
| Lots | 3 | 0.4015 | $1.338X10^{-1}$ | 34.04 | $1.899X10^{-4}$ | 46.2 | 13.78 | 3.29 |
| Rotors within lots | 8 | 0.03145 | $3.931X10^{-3}$ | 8.045 | $1.510X10^{-5}$ | 3.7 | 3.886 | 0.93 |
| Cuvettes within rotors | 216 | 0.1055 | $4.886X10^{-4}$ | 94.36 | $4.028X10^{-5}$ | 9.8 | 6.347 | 1.51 |
| Readings within cuvettes | 2508 | 0.01299 | $5.178X10^{-6}$ [a] | | $1.657X10^{-4}$ [b] | 40.3[c] | 12.87[c] | 3.07[c] |
| Total | 2735 | 0.5515 | | | | 100.0 | | |

*[a] Variance of the mean of 32 photometric readings averaged to report one absorbance value.*
*[b] Variance of any one of the 32 photometric readings averaged to report one absorbance value. Computed as 32 x the Mean Square.*
*[c] For a single one of the 32 photometric readings averaged to report one absorbance value.*

**Table 4. Three-level nested, balanced design ANOVA – 3 Lots.**

| Source | df | Sum of squares | Mean square | Mean square ratios | Variance | % of total variance | Standard deviation | CV(%) |
|---|---|---|---|---|---|---|---|---|
| Lots | 2 | 0.1987 | $9.936X10^{-2}$ | 19.54 | $1.378X10^{-4}$ | 68.6 | 11.74 | 2.83 |
| Rotors within lots | 6 | 0.03051 | $5.086X10^{-3}$ | 12.29 | $2.049X10^{-5}$ | 10.2 | 4.53 | 1.09 |
| Cuvettes within rotors | 162 | 0.06704 | $4.138X10^{-4}$ | 166.0 | $3.446X10^{-5}$ | 17.2 | 5.87 | 1.42 |
| Readings within cuvettes | 1881 | 0.000469 | $2.493X10^{-7}$ [a] | | $7.978X10^{-6}$ [b] | 4.0[c] | 2.82[c] | 0.68[c] |
| Total | 2051 | 0.2968 | | | | | 100.0 | |

*[a] Variance of the mean of 32 photometric readings averaged to report one absorbance value.*
*[b] Variance of any one of the 32 photometric readings averaged to report one absorbance value. Computed as 32 x the Mean Square.*
*[c] For a single one of the 32 photometric readings averaged to report one absorbance value.*

equation predicts an $s_{\bar{x}}$ of 0.14 mA. Squaring $s_{\bar{x}}$ and multiplying by 30 (the number of readings he averaged to report one value) yields a variance for a single photometric sampling of $5.8 \times 10^{-7}$ (SD = 0.8 mA). Again, the variance quoted for the GEMSAEC is about an order of magnitude lower than that which we compute for our instrument. In contrast to the Multistat III, the GEMSAEC does not use a disposable rotor and does perform an initial correction on each absorbance reading in an attempt to reduce the cuvette-to-cuvette variability. By using only the three rotor lots for the instrument at an absorbance of 0.4144, an SD of 2.8 mA for a single measurement (CV = 0.7%) was calculated. The CV's computed for the same absorbances for the instruments of Tiffany and Maclin are about 0.2%. In this case, it is obvious that the lot of rotors which were eliminated severely affected the estimates of the precision of a single measurement because that lot contained rotors which gave highly imprecise absorbance readings. The data shown in Table 4 are much more representative of the actual precision attainable with the instrument in this laboratory.

REFERENCES

[1] Pardue, H.L. Hewitt, T.E., and Milano, M.J., *Clinical Chemistry,* 1974, **20,** 1028.
[2] Maclin, E., Rohlfing, D., and Ansour, M., *Clinical Chemistry,* 1973, **19,** 832.
[3] Ingle, J.D. Jr, and Crouch, S.R., *Analytical Chemistry,* 1972, **44,** 1375.
[4] Ingle, J.D. Jr, and Crouch, S.R., *Analytical Chemistry,* 1973, **45,** 333.
[5] Tiffany, T.O., Thayer, P.C., Coelho, C.M., and Manning, G.B., *Clinical Chemistry,* 1976, **22,** 1438.
[6] Ingle, J.D. Jr, and Crouch, S.R., *Analytical Chemistry,* 1972, **44,** 785.
[7] Ingle, J.D. Jr, *Analytical Chemistry,* 1973, **45,** 861.
[8] Maclin, E., *Clinical Chemistry,* 1971, **17,** 707.
[9] Hewitt, T.E., and Pardue, H.L., *Clinical Chemistry,* 1975, **21,** 249.
[10] Talmi, Y., Crosmun, R., and Larson, N.M., *Analytical Chemistry,* 1976, **48,** 326.
[11] Instrumentation Guidelines Study Group, Subcommittee on Enzymes, Committee on Standards, American Association for Clinical Chemistry, *Clinical Chemistry,* 1977, **23,** 2160.
[12] National Committee for Clinical Laboratory Standards, Tentative Standard: TSI-3. Standard for Determining Spectrophotometer Performance Criteria. National Committee for Clinical Laboratory Standards, Villanova, PA 19085. 1978.
[13] Bowers, G.N., Jr., Bergmeyer, H.U., Hørder, M., and Moss, D.W., *Clinica Chimica Acta,* 1979, **98,** 163F.
[14] American Society for Testing and Materials. "Manual on Recommended Practices in Spectrophotometry". 3rd edition. 1969. American Society for Testing and Materials. Philadelphia, PA.
[15] Mendenhall, William, "Introduction to Linear Models and the Design and Analysis of Experiments", 1968. Duxbury Press, Belmont, CA. Chapter 12.
[16] Ostle, Bernard, "Statistics in Research", 1963. Iowa State University Press, Ames, IA, 1963, p.70.
[17] Ostle, Bernard, "Statistics in Research", 1963. Iowa State University Press, Ames, IA, 1963, p.80.

DISCLAIMER

*The use of trade names is for identification only and does not constitute endorsement by the Center for Disease Control, by the Public Health Service, or by the U.S. Department of Health and Human Services.*

